# Methylene Blue Effectiveness as Local Analgesic after Anorectal Surgery: A Literature Review

**DOI:** 10.1155/2017/3968278

**Published:** 2017-08-15

**Authors:** Dewi Fransiska, Wifanto Saditya Jeo, Yefta Moenadjat, Dewi Friska

**Affiliations:** ^1^Surgery Department, Faculty of Medicine, Universitas Indonesia, Jakarta, Indonesia; ^2^Community Medicine Department, Faculty of Medicine, Universitas Indonesia, Jakarta, Indonesia

## Abstract

**Background:**

Methylene blue (MB) has been found to have unique analgesic property through temporary disruption of sensory nerve conduction. In anorectal surgery, MB is widely used as a biologic stain but the analgesic effect has never been studied. Thus, a literature review completed with critical appraisal is required to find out its efficacy.

**Methods:**

A review has been run to find out its efficacy. Literature search proceeded in database sites, namely, PubMed, EBSCO, Cochrane, Wiley, and ProQuest using the following keywords: “anorectal” OR “hemorrhoid” OR “anal fistula” OR “anal fissure” OR “anal abscess” OR “anal pruritus” AND “methylene blue” AND “analgesic”; then the critical appraisal and its implication were discussed.

**Result:**

There were 491 articles in full text found, and four studies met the inclusion criteria. Two studies were focused on the evaluation of VAS in hemorrhoid surgery whereas the rest were focused on the evaluation of symptom score in anal pruritus.

**Conclusions:**

A study with level of evidence 2 on VAS showed the efficacy. The rest showed insufficient evidence due to variations of anorectal surgery and the methods and techniques of MB application. A further prospective clinical study is required.

## 1. Introduction

Nowadays, methylene blue (MB) is widely used as biologic stain. In anorectal surgery, the use of high concentrated topical MB is not only staining the tip and nerve fibers, but also disrupting their function temporarily. To this knowledge, MB has been used in the treatment of neuritis, to reduce pruritus and analgesia in anorectal surgery [1–3].

Pain and itch receptors are nerve endings that are not myelinated, located in the papillary layer of the skin with the highest numbers being in the epidermis and dermis. When this receptor is stimulated, excitatory neurons transmitted impulses to the dorsal horn of spinal cord and then continued to central nervous system to create pain or itch sensation [3–5]. This mechanism explains the efficacy of MB to reduce pain and itch.

Subjects with perianal fistula where the tract is injected with MB will have lower postoperative pain compared to those who were not injected with it [6]. Studies in Singapore and China showed that perianal intradermal injection of MB provides temporary pain relief after hemorrhoidectomy and lateral anal sphincterotomy [1, 6–8]. Other studies showed that subjects with severe anal pruritus (intractable anal pruritus) who were injected with MB intradermally had better symptom score [9, 10].

However, published information of the efficacy in anorectal surgery remains unclear. Thus, a literature review was run, aimed at finding out its efficacy.

## 2. Method

Literature search was run with clinical questions based on PICO: population was those having anorectal surgery, the intervention was the application of MB, the comparison was to those who were not injected with MB, and the outcome was decrease of pain or symptom score. Literature search proceeded in database sites, namely, PubMed, EBSCO, Wiley, Cochrane, and ProQuest with clinical question “Does application of MB post anorectal surgery can give analgesic effect?” and keywords “anorectal” OR “hemorrhoid” OR “anal fistula” OR “anal fissure” OR “anal abscess” OR “anal pruritus” AND “methylene blue” AND “analgesic”. Inclusion criteria for literature research were English or* Bahasa* language, full text, and publication in recent ten years. Exclusion criteria were congenital disease, autoimmune, malignancy, subject with age under 12 years, molecular study, animal study, short communication, editorial, and commentary letter. Critical appraisal of validity, importance, and applicability was addressed based on Oxford critical appraisal tools which can be downloaded from http://www.cebm.net/critical-appraisal/.

## 3. Result

**Figure 1:**
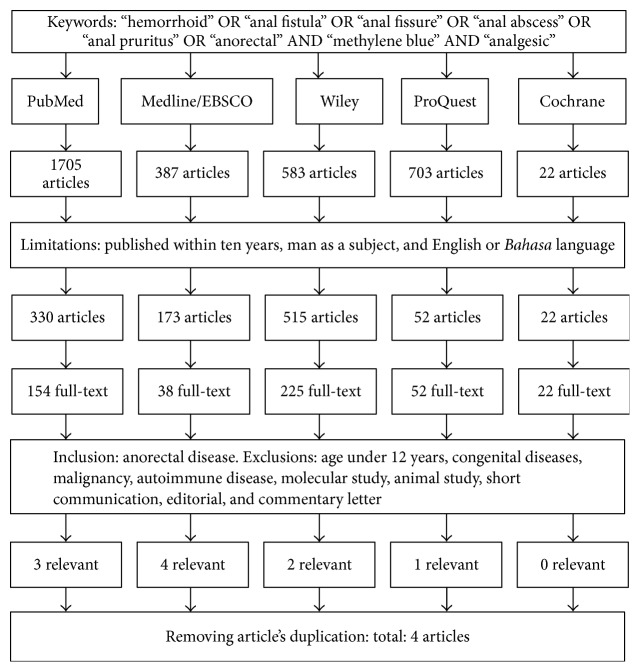
Diagram of the steps of literature search.

On literature searching based on the steps shown in a diagram explained in Figure 1, there were 4 studies that met the criteria and were summarized in Table 1.

## 4. Discussion

All the studies included showed different subjects' characteristics and methods of MB applications. Xiang and Feng and Sim and Tan focused on hemorrhoid while another two studies focused on anal pruritus. In the studies on hemorrhoid, evaluation was addressed to VAS pain score, while as in anal pruritus the evaluation was using degree of symptom score. Study of Xiang and Feng which was a case control study showed low level of evidence (LOE 4). In contrast, study of Sim and Tan which was a randomized single-blind clinical trial showed a higher level of evidence (LOE 2).

In the study of Xiang and Feng, subcutaneous MB and ropivacaine solution was treated as group of therapy and conventional therapy (consisting of tramadol 100 mg orally twice daily for five days) was treated as control group [6]. Sim and Tan applied intradermal injection of MB and Marcain solution in the treatment group, compared to intradermal injection of Marcain and saline solution in control group [1]. Both of the studies assessed degree of VAS reduction after hemorrhoidectomy that was observed for 14-day period on 151 samples (Xiang and Feng) and 67 samples (Sim and Tan) [1, 6].

The characteristics of age, sex, degree of hemorrhoids, and the number of incisions in Xiang and Feng study were not significantly different (*p* = 0.128; *p* = 0.7; *p* = 0.894; *p* = 0.774) [6]. In Sim and Tan's study, the *p* values of patients' age, genders, hemorrhoid thrombosis, hemorrhoid bleeding, hemorrhoids number, length of operating time (minutes), and hospitalization stays of 67 patients were 0.281, 0.112, 0.6, 0.424, 0.915, 0.754, and 0.931 [1]. Based on these data we can conclude that there is no statistically significant difference in demographic and clinical characteristics in the study of Sim and Tan.

Xiang and Feng compared VAS values and postoperative complications between the treatment and control group. There are statistically significant differences of VAS in postoperative day from the first until the third day (*p* < 0.01) and the fourth until the fifth day (*p* < 0.05), whereas there is no significant difference of the VAS values in the sixth until the 14th postoperative day (*p* > 0.05) [6]. Complications such as skin necrosis and skin infections were not be found in each group, while complications of uroschesis, crissum skin abnormality, temporary anal incontinence, and edema in the incision area are not significantly different in the two test groups (*p* values of 0.784, 0.459, 0.691, and 0.651, resp.) [6].

In Sim and Tan's study, there is significant difference clinically in VAS score from the first to third postoperative day (*p* = 0.026), but there is no significant difference after the third to the 14th days in both groups [1]. No differences are significant in postoperative VAS that require the patient to go for control behind his schedule (*p* = 0.448) and the need for rehospitalization (*p* = 0.421) [1]. In addition, complications such as acute urinary retention, secondary bleeding, pruritus, and temporary incontinence also do not have significant differences in both groups (*p* values of 0.197, 0.552, 0.699, and 0.552, resp.) [1]. Another complication in the form of local skin reactions cannot be found in both groups.

In Xiang and Feng's study, relative risk (RR) of MB injection to prevent increasing VAS for three days postoperatively was 57.4%. It correlates with the results of number needed to treat (NNT) where it takes four patients to get MB injection to prevent one complication with confidence interval (CI) as follows: 95% CI 2.63–13.16. In addition, RR to prevent increasing VAS for five days postoperatively was 64%. This is consistent with the results of NNT where it takes eight patients to receive MB injection to prevent complications with 95% CI 3.597–27.778.

In Sim and Tan's study, postoperative MB injection can prevent increasing VAS for three days as much as 73% (RR = 0.27). This was found to be correlated with NNT where it takes 14 patients to get MB injection to prevent complications with 95% CI 5.21 to 21.74. There is a 2.3-fold increasing risk of acute urinary retention in the group not injected with MB with 95% CI 1.754 to 3.067.

Based on the two studies above, injection of MB effectively decreases VAS from the first to the third day postoperatively. Nevertheless, postoperative VAS after the fourth day was found different in the two studies above.

In contrast to the above studies, Samalavicius et al. used intradermal injection of 1% MB in ten idiopathic pruritus ani (IPA) patients, while Sutherland et al. used intradermal injection of MB, Marcain, and adrenaline solution in 49 refractory pruritus ani (RPA) patients to assess the effectiveness of the MB in reducing symptom score [9, 10]. Both studies had a low level of evidence (LOE 4) as a prospective case series.

In Samalavicius et al.'s study, ten IPA patients (six men and four women) were followed up prospectively every six months, every 12 months, and every year (median is 47 months with range being 29–60 months) [9]. There are eight patients who experienced recurrence where the fastest emerging symptom happened in one patient within two months after intradermal MB injection and the symptom score is four. All of them do not need repeated MB injections and their complaints can be solved with conservative therapy [9].

Sutherland et al. prospectively evaluated symptom score and complications after MB injection in 49 patients with RPA (30 females and 19 males) who were treated by one surgeon (FAF) [10]. Median age of patients was 43 years with range 19–67 years. Follow-up was conducted over four and eight weeks. Of the 19 patients who experienced relapse, four patients are given a second injection of MB with improved symptom scores of five in all patients [10].

All patients in the study of Samalavicius et al. do not have complications such as skin necrosis, anaphylactic reactions, and changes in fecal continence [9]. In the study of Sutherland et al., seven patients had fecal continence changes but there is improvement on the tenth day and sixth week. Two patients had perianal sensation disorders, whereas skin necrosis and anaphylactic reactions were not found [10].

Even though those studies showed efficacy of the use of MB, it should be noted that those studies proceeded using a small sample. It is believed that finding “strong” evidence is difficult due to the diversity of anorectal disorders and method of application of MB.

## 5. Conclusion

MB was shown to be effective in reducing degree of pain (VAS) with level of evidence 2 which is quite strong and effectively reduce anal pruritus (level of evidence 4), which is weak. Somehow, this review may initiate further study on the use of MB as a local analgesic for postoperative management of those who had anorectal surgery.

## Figures and Tables

**Table 1:** Critical appraisal of the studies.

Articles	Years	Validity							Relevance		
		Study design	Number of patients	Randomization	Similarity treatment & control	Blinding	Comparable treatment	Intention to treat	Determinant	Measurement of outcome	Level of evidence
Xiang and Feng [6]	2015	Retrospective study (case-control)	151	−	+	−	+	−	+	+	4
Sim and Tan [1]	2013	Randomized single-blind clinical trial	67	+	+	+	+	−	+	+	2
Samalavicius et al. [9]	2012	Prospective study (case series follow-up)	10	−	+	−	−	−	−	+	4
Sutherland et al. [10]	2009	Prospective study (case series follow-up)	49	−	+	−	−	−	−	+	4
